# Predictivity of Antemortem Findings on Postmortem Inspection in Italian Heavy Pigs Slaughterhouses

**DOI:** 10.3390/ani11082470

**Published:** 2021-08-23

**Authors:** Sergio Ghidini, Giovanni Loris Alborali, Silvio De Luca, Antonio Marco Maisano, Federica Guadagno, Mauro Conter, Adriana Ianieri, Emanuela Zanardi

**Affiliations:** 1 Department of Food and Drug, University of Parma, Via del Taglio 10, 43126 Parma, Italy; sergio.ghidini@unipr.it (S.G.); adriana.ianieri@unipr.it (A.I.); emanuela.zanardi@unipr.it (E.Z.); 2 Headquarters, Istituto Zooprofilattico Sperimentale della Lombardia e dell’Emilia Romagna, Via A. Bianchi 9, 25124 Brescia, Italy; giovanni.alborali@izsler.it (G.L.A.); federica.guadagno@izsler.it (F.G.); 3 Territorial Section of Lodi, Istituto Zooprofilattico Sperimentale della Lombardia e dell’Emilia Romagna, Via A. Einstein, 26900 Lodi, Italy; antoniomarco.maisano@izsler.it; 4 Independent Researcher, 43100 Parma, Italy; missgazzella@gmail.com

**Keywords:** meat inspection, slaughterhouse, pig health monitoring, antemortem, postmortem, heavy pigs

## Abstract

**Simple Summary:**

Inspections of pigs before (antemortem) and after (postmortem) being slaughtered are part of the official controls carried out in European abattoirs. The ability of data obtained from the antemortem inspections to predict lesions eventually found during postmortem inspections has not been thoroughly investigated so far. In this study, data obtained from inspections performed both ante- and postmortem in heavy pigs slaughtered in Italy were analyzed, determining the prevalence of the most common lesions and conditions found during the ante- and postmortem inspections and exploring the correlation between these findings. The most common findings were the presence of manure on more than the 30% of the body and pleurisy for antemortem and postmortem inspections, respectively. Some conditions found during the antemortem inspections were predictive of lesions reported during postmortem inspections. For instance, respiratory and kidney lesions were more likely to occur in pigs presenting manure on more than the 30% of the body, whereas dermatitis and skin wounds were more likely to be present in pigs showing skin lesions during the antemortem inspections. The results of this study show that information obtained from the antemortem inspection of pigs can be useful to characterize farms using a risk-based approach and to address the organization of official controls in slaughterhouses.

**Abstract:**

Pigs slaughtered in European abattoirs must be submitted to antemortem inspection (AMI) and postmortem inspection (PMI), as required by the current European legislation in the matter of official controls. AMI and PMI are equally essential to guarantee food safety and to monitor swine health and welfare. However, little is known about the ability of AMI to predict conditions that are possibly found during PMI. In this study, such a correlation was explored together with the assessment of conditions typically found during AMI and PMI in heavy pigs slaughtered in two Italian slaughterhouses. An assessment scheme containing 13 variables for AMI and 34 lesions for PMI was used for the scope. The herd size was also considered as a variable and included in the study. A total of 24,510 pigs and 30,961 pigs were assessed during AMI and PMI, respectively. The most common conditions found were manure on the body covering more than 30% of the body (dirt >30%) and pluck lesions (‘pleurisy’, ‘pericarditis’, and ‘pneumonia’) for AMI and PMI, respectively. A significant correlation (*p* < 0.05) between some antemortem (AM) findings and postmortem (PM) conditions was found. In particular, the AM conditions ‘dirt >30%’and ‘skin lesions’ were positively related with PM conditions ‘skin wounds’ and ‘dermatitis’, while the complexes of respiratory and kidney lesions were predicted only by the condition ‘dirt >30%’. The variable ‘standardized herd size’ was negatively associated with ‘milk spot liver’ and positively associated with ‘arthritis/bursitis’. The results of this study show that findings reported during AMI can potentially be used to predict certain conditions found in pigs at PMI. These data can be useful for the competent authorities in characterizing swine farms using a risk-based approach and in developing systems and specific plans for official controls.

## 1. Introduction

Meat inspection (MI) activities carried out in slaughterhouses serve different purposes. Primarily born with the aim of protecting consumers from foodborne hazards and ensuring food safety and quality [1], MI activities have recently broadened their scope, particularly including the monitoring of animal health and welfare status [2]. In Europe, MI is regulated by rules laid down in Regulation (EU) 2017/625 of the European Parliament and the Council [3] and in the Commission Implementing Regulation (EU) 2019/627 [4]. MI tasks encompass a series of activities planned according to a risk-based approach and carried out at the slaughterhouse by the Competent Authority (CA) of each Member State.

These activities are set before and after the stunning/death of the animals, and some of them are constituted by ante and postmortem inspections (AMI and PMI) [5]. With regard to pigs, there are a number of published studies that mainly focused on lesions derived from PMI rather than AMI at the European [6,7,8,9] and Italian level [10,11,12]. The data collected at the slaughterhouse during PMI are certainly of great importance because they may be indicatives of some diseases or of not optimal welfare [13]. However, results from AMI can contribute to several aspects concerning pig health and welfare, as well suggest the actions that should be undertaken when certain conditions are met at the abattoir. In fact, although PMI in pigs is only visual in European slaughterhouses [10], unless differently specified by procedures required for exporting meat and meat products in non-EU countries [14], the official veterinarians (OVs) can decide regarding additional procedures such as palpation and incision of organs in cases of a suspected risk for public health, animal health, or animal welfare during the AMI [15]. Therefore, operations performed during AMI may help OVs in identifying the batches of pigs that are not suitable for visual-only inspection and that require more thorough inspection procedures [16]. Pigs not suitable for visual-only inspection also might require optimization of slaughter and inspection procedures, such as moving carcasses that need additional inspection to a separate line, a reduction in the line speed, and an increased number of operators on the trimming line [16].

In order to apply such measures, both OVs and food business operators (FBOs) need specific and reliable indicators that can facilitate the decision-making process. Little is known concerning the relationship between findings reported during AMI and those found during PMI in pig abattoirs [17]. To the best of our knowledge, a determination of the predictive value of certain conditions present during AMI with respect to lesions assessable during PMI in slaughtered pig has not yet been performed in heavy pigs. The aim of this study was to assess such a correlation in heavy pigs slaughtered for protected designation of origin (PDO) production in two Italian slaughterhouses. Moreover, the prevalence of the AMI and PMI findings was established and compared to the data previously reported in the literature.

## 2. Materials and Methods

### 2.1. Data Collection

Data were collected in two commercial abattoirs located in the two Italian regions, Lombardy and Emilia-Romagna, where around the 60% of the Italian pig population was located [18]. Both establishments had a weekly output of about 10,000–10,500 pigs, for a total of about 960,000 pigs slaughtered per year considering both premises, representing around 8.3% of the total amount of pigs slaughtered per year at a national level [18]. The two slaughterhouses received heavy pigs from farms located in the two regions at 160–170 kg live body weight and 9 months of age destined for PDO production and fresh meat products. The animals were purebred pigs of the basic traditional Large White and Landrace breeds or animals derived from those breeds. The two abattoirs were under EU legislation on animal welfare at the time of the study. The research was performed during a 3 month period between August and October 2018. A scheme based on visual inspection was used to assess specific lesions and conditions during both ante- and postmortem evaluation; such a scheme was already included into ‘CLASSYFARM’, an information system implemented by the Italian Ministry of Health that allows categorization of farms according to the risk [19]. In Table 1, the guidelines for the correct reporting of lesions and conditions included in this study are described.

The assessment of antemortem and postmortem conditions was performed by two expert veterinarians trained at the beginning of the study to ensure agreement between observers. The training consisted of 10 whole-day scoring sessions performed directly at both slaughterhouses (5 days per each abattoir) under the supervision of S.G. The two investigators alternatively performed AMI or PMI, switching their positions every 2 h during the sampling day. The antemortem inspections were performed during the unloading process of the pigs from the trucks. In particular, the pigs were directed from the trucks toward the pens in the lairage through a corridor. Before entering the pen, an operator present at this point reduced the speed of the pigs and increased the spaces between them using a red sorting paddle, allowing one of the two investigators to evaluate the presence of lesions and other conditions (e.g., the degree of dirtiness) on a side of the pigs (Table 1). This was a standard procedure carried out in both abattoirs to reduce the chances of injuries when the pigs were to enter the pens in the lairage. The antemortem assessment was performed at a distance of about 1 m from the abovementioned corridor prior to entering the pen, while the postmortem inspections were executed at the inspection points beside the official veterinarians carrying out official controls. Batches of pigs were randomly selected during the day of data sampling. The sampling unit considered in our study was the batch; hence, the batches from AMI to PMI were followed through the identification mark located on the shoulder as required by the current legislation [20]. Pigs were not individually followed during AMI and PMI because of the mixing process carried out routinely after the unloading and lairage of the pigs before slaughtering. The AMI inspections were performed on all pigs unloaded from the truck when arriving at the two slaughterhouses; however, in some cases, the investigators were not able to assess all the pigs unloaded for the trucks due to technical and safety reasons (e.g., the assistants were not able to reduce their speed when entering the pen). If the number of pigs present in AMI was lower than 70% of the same batches assessed during the PMI, these were excluded from the analysis.

Consequently, only batches of pigs presenting both AMI and PMI data were analyzed, with a total of 182 batches coming from 98 farms included in the study. Moreover, the information regarding the herd size (number of pigs raised/farm/year) was extrapolated from the Food Chain Information (FCI) form and included as a variable. The results were first reported on paper and then transferred to a Microsoft Excel spreadsheet for further analysis.

### 2.2. Statistical Analysis

Firstly, through a mixed Poisson regression, the association between the total number of postmortem conditions recorded in a batch and the corresponding number of observed antemortem lesions (no. of total antemortem lesions/no. of examined pigs) was investigated. The count of postmortem lesions in each batch was used as the response variable and the ln-transformed batch size was included as an offset variable. In this and in all further models, standardized herd size ((*x* − mean)/SD) was included as a covariate, and farm was added as a random residual factor with a compound symmetry covariance structure, to account for potential correlation among batches from the same farm.

A second Poisson model was carried out at batch level to assess the relationship between the total count of postmortem lesions and the recorded proportion of selected antemortem lesions. Focus was placed on the most common lesions (i.e., observed in over 20% of batches), thus including, as explanatory variables, the within-batch proportions of dirt >30%, skin lesions, ear lesions, lameness, umbilical hernia, and suppressed animals. Lastly, the association between the probability of observing a specific postmortem variable and the proportion of the abovementioned antemortem variables was assessed at batch level through a set of mixed logistic regressions. Once again, focus was put on the most frequent lesions, using as response variables in separate binomial models the recorded proportions (no. of events/batch size) of respiratory lesions (i.e., the sum of pleurisy and pneumonia), pericarditis, skin wounds, milk spot liver, liver alterations, dermatitis, arthritis/bursitis, kidney lesions (i.e., the sum of nephrosis and nephritis), abscesses, and enteritis/colitis.

In all cases, full models were first fitted, and then minimal models were obtained through backward elimination of nonsignificant variables (partial *p*-value for removal set at 0.15). Significance level was set at 0.05 and, unless otherwise indicated, results are presented as the mean ± standard error (SE). All analyses were carried out through PROC GLIMMIX in SAS/STAT 9.4 software (Copyright © 2021, SAS Institute Inc., Cary, NC, USA).

## 3. Results

### 3.1. Descriptive Results

The study population included inspection data on 182 batches from 98 different farms. Antemortem lesions were recorded on a total of 24,510 pigs (mean ± SE: 134 ± 2 animals/batch; range: 52–272), while postmortem data were collected from 30,961 animals (mean ± SE: 169 ± 5 animals/batch; range: 50–457) from the same batches. Herd size was highly variable, with a mean of 5606 ± 323 SE pigs/farm (range: 139–16,838).

**Figure 1:**
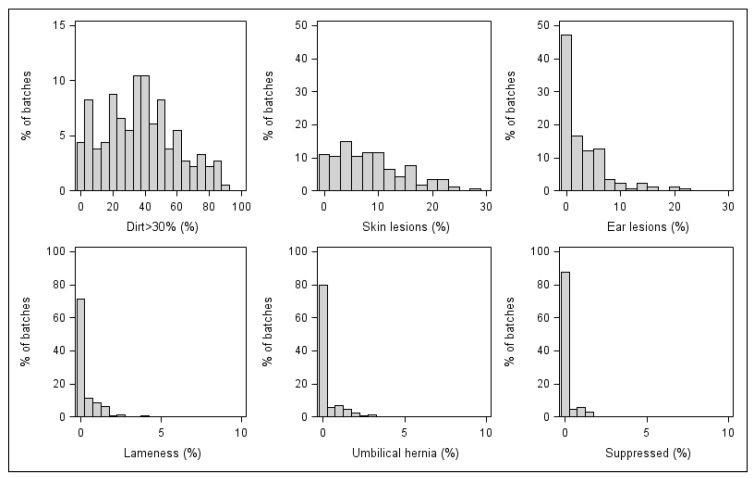
Frequency distribution of within-batch prevalence of antemortem lesions recorded in slaughtered pigs (*N* = 182 batches).

Overall, the most common antemortem conditions were dirt >30%, with at least one recorded pig in 177/182 (97.2%) batches, and skin lesions, observed in 167/182 (91.8%) batches. Dirt >30% was also the most prevalent record within batches, with a mean prevalence of 37.1% (95% CI: 33.8–40.4%). Batch-level descriptive data of all recorded antemortem lesions are detailed in Table 2, and frequency distributions of prevalence for the most common lesions are shown in Figure 1.

**Figure 2:**
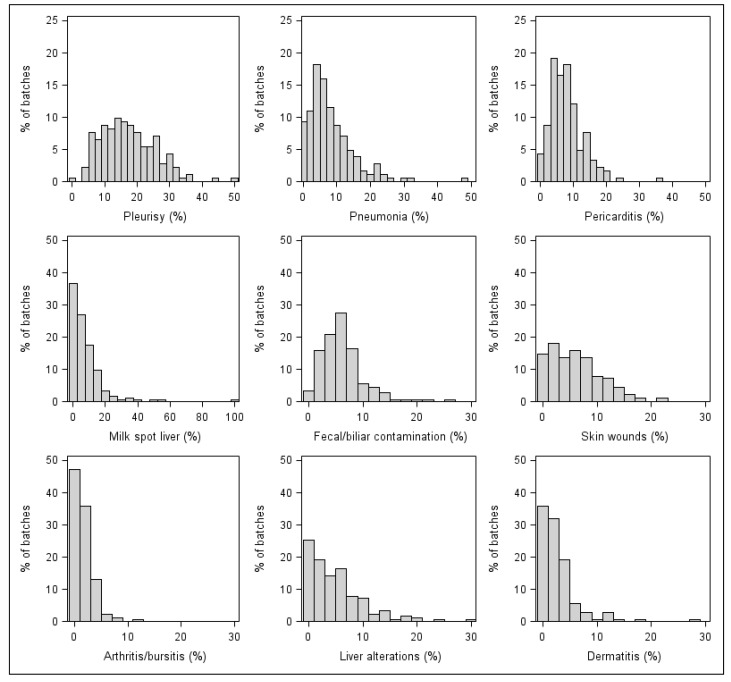
Frequency distribution of within-batch prevalence of postmortem lesions recorded in slaughtered pigs (*N* = 182 batches).

The most frequently observed postmortem lesions were those related to respiratory diseases, with at least one recorded pig per batch suffering from either pleurisy or a type of pneumonia. They were followed by pericarditis, observed at least once in 177 (97.2%) out of 182 batches. Pleurisy was the most prevalent lesion within batches, with a mean value of 17.2% (95% CI: 16.0–18.4%). Descriptive data of all recorded postmortem lesions are reported in Table 3, while frequency distributions of prevalence for the most common lesions are shown in Figure 2.

### 3.2. Relationship between Ante- and Postmortem Lesions

In general, the total count of postmortem lesions increased significantly with the number of observed antemortem lesions (parameter estimate ± SE: 0.67 ± 0.11; χ^2^_1_ = 36.04; *p* < 0.0001). In particular, the most influential lesions were dirt >30% (*p* = 0.0003) and skin lesions (*p* = 0.0072).

Concerning the probability of observing specific postmortem lesions, respiratory diseases were positively related with dirt >30% (*p* = 0.002, Table 4), with a 10% increase in the recorded proportion of dirty pigs leading to a 7% increase in the odds of observing respiratory lesions at postmortem (OR = 1.07; 95% CI: 1.02–1.11).

Skin wounds were positively associated with the proportion of skin lesions and dirt >30% recorded during the AMI (*p* = 0.031 and *p* = 0.045, respectively). A 10% increase in skin lesions led to a 14% increase in the odds of observing skin wounds (OR = 1.14; 95% CI: 1.01–1.28), while the same increase in dirt >30% led to a 5% increase (OR = 1.05; 95% CI: 1.01–1.11). Similarly, dermatitis was positively associated with skin lesions and dirt >30% (*p* = 0.0002 and *p* = 0.0031, respectively). In detail, a 10% increase in the proportion of dirty pigs in a batch led to a 11% increase in the odds of observing dermatitis postmortem (OR = 1.11; 95% CI: 1.03–1.20); the same increase in the proportion of pigs showing skin lesions at antemortem inspection led to a 35% increase in the odds of pigs from that batch showing dermatitis (OR = 1.35; 95% CI: 1.18–1.54).

The odds of observing milk spot liver in pigs were negatively related to herd size (*p* = 0.0002), but positively related to the proportion of skin lesions recorded at antemortem inspection (*p* = 0.02). An increase in herd size of 500 pigs resulted in a 6% decrease in the odds of observing a milk spot liver (OR = 0.94; 95% CI: 0.91–0.97), while a 10% increase in skin lesions resulted in a 24% odds increase (OR = 1.24; 95% CI: 1.03–1.49). The odds of detecting arthritis or bursitis postmortem were instead affected positively by herd size (*p* = 0.0064), with a 500 pig increase leading to a 1.5% odds increase (OR = 1.015; 95% CI: 1.004–1.026).

Kidney lesions were associated only with dirt >30% (*p* = 0.002); a 10% increase in the proportion of dirty pigs resulted in an 18% increase (OR = 1.18; 95% CI: 1.06–1.32) in the odds of observing nephrosis or nephritis. Abscesses were positively associated with the proportion of observed lameness (*p* = 0.0007) and ear lesions (*p* = 0.0041). A 1% increase in the proportion of lameness resulted in a 42% increase in the odds of observing abscesses (OR = 1.42; 95% CI: 1.16–1.74), while a 10% increase in ear lesions led to a 32% odds increase (OR = 1.015; 95% CI: 1.09–1.60).

Pericarditis and liver alterations were not affected by any of the examined antemortem variables (all *p* > 0.05).

## 4. Discussion

### 4.1. Antemortem Findings

With regard to the antemortem findings, manure on the body and skin lesions were the most frequently observed conditions with a mean within-batch prevalence of 37.13% and 9.07%, respectively.

Evaluation of the dirtiness in pigs is generally considered an indicator of animal welfare with regard to the ‘good housing’ principle described by the Welfare Quality protocol for fattening pigs [21,22]. In fact, such a finding, together with bursitis, can reflect the effect of the different floors used in pig farms [23], although the cleanliness of pigs submitted to antemortem inspection can be influenced by other factors such as the transport and the lairage [24]. Similar findings were reported in another study performed in an Italian slaughterhouse, with more than 40% of 10,085 heavy pigs submitted to antemortem inspection presenting dirtiness on more than the 20% of the body [24].

Skin lesions are also considered good indicators on animal welfare at the abattoir [2]. As a matter of fact, skin lesions can predict the welfare status of pigs with damage occurring up to 11 weeks prior to slaughtering [25]. However, similarly to the presence of manure on the body, skin lesions can be influenced by several factors not necessarily occurring on the farm, such as mixing before loading, transport, and lairage [26,27].

Following dirtiness and skin lesions, ear lesions were the third most frequently observed condition, with a mean within-batch prevalence of 3.30%. Ear lesions, together with tail-biting lesions and lameness, are considered indicators of ‘good health’ in the context of principles stated by the Welfare Quality protocols for fattening pigs [22]. The prevalence of ear lesions found in this study was similar to the prevalence reported by Maisano et al. [24], but lower compared to Bottacini et al. [28], with a reported prevalence of 9.0% of ear lesions in heavy pigs; these differences can be attributed to differences in the breeding conditions of the pigs (e.g., floor types or environmental enrichment) or in the assessment of the considered lesions.

Both lameness and tail-biting lesions resulted with a low prevalence in this study. These findings are similar to those reported by Maisano et al. [24], reporting a prevalence of 0.3% of animal presenting lameness, while tail-biting lesions were never detected. Tail lesions are generally considered ‘iceberg’ indicators of animal welfare; this definition describes an indicator that can alone provide an overall assessment of the welfare status [29]. The importance of these lesions is also provided by their correlation with reduced cold carcass weight and increased rate of total and partial condemnation rate [30,31]. However, all the pigs inspected in this study were tail-docked; the prevalence of tail lesions in pigs at slaughterhouse can be strongly influenced by tail docking, being more easily detected in pigs with intact tails [32,33].

In this study, in seven of the 182 analyzed batches, pigs were found dead during the transport (‘dead on arrival’), while, in 23 of the 182 analyzed batches, pigs reported lesions that required suppression in the pen (‘suppressed’), respectively. These findings emphasize the importance of the transport of the pigs to the slaughterhouse, which represents a critical point in the pig production chain [34]. The mortality due to transport can be considered an appropriate indicator of the welfare during the transport for pigs. The transport mortality rate depends on several factors; among these, temperature stress plays an important role. Guardone et al. [11] reported a higher rate of death during transport in August, September, and December. In these months, the temperature is normally out of the thermal neutral range for pigs (15–25 °C).

### 4.2. Postmortem Findings

The most lesions reported during postmortem inspections were respiratory lesions, with pleurisy (17.21%) and pneumonia (8.16%) as the first and second most frequently reported conditions, respectively (Table 3). This finding is not surprising, considering the critical impact that respiratory diseases involving primarily *Mycoplasma hyopneuomoniae* and *Actinobacillus pleuropneumoniae* has on the pig industry [35]. Ghidini et al. [10] reported similar findings, with 15.46% and 6.43% of pigs examined from three Italian slaughterhouses showing pleuropneumonia and pneumonia, respectively. Similarly, Maisano et al. [24] reported a high prevalence of pleurisy (25.78%) and pneumonia (17.09%) in heavy pigs slaughtered for PDO production. On the other hand, Guardone et al. [11] found a lower prevalence of these lesions, with 6.9% and 3.5% of pleuropneumonia and pneumonia reported, respectively. Even lower prevalence was described by Ceccarelli et al. [12], with only 0.12% of lungs condemned for polyserositis in their study. Such differences can be explained by variations in the methods used to identify the lesions, farm management, and interobserver disagreement [36]. Pericarditis was also often reported in this study, with 7.92% of within-batch prevalence. This observation rate was slightly higher than the prevalence of pericarditis indicated in the studies of Guardone et al. [11] (6.94%), Maisano et al. [24] (4.25%), and Ghidini et al. [10] (3.22%), while Ceccarelli et al. [12] reported a higher percentage (10.77%) of heart conditions, mainly for pericarditis.

Lesions affecting the liver were also frequently detected in this study, with a mean within-batch prevalence of milk spot lesions and liver alterations of 7.60% and 4.89%, respectively. Milk spot lesions are frequently reported in the literature as a major cause of liver condemnation, although the prevalence of this condition may vary greatly among studies [10,37]. In a recent retrospective study evaluating the cause of partial and total condemnation in pig slaughterhouses, it was estimated that this condition can cause up to 96.2% of the total liver condemnations [12]. Maisano et al. [24] reported a higher prevalence of milk spot lesions (25%), similar to the results of Scollo et al. [38] reporting a prevalence of livers with severe lesions of 23.93%. On the other hand, other studies have reported a lower prevalence of milk spot lesions. For instance, Konsted and Kørensten [9] described a prevalence of 4.6% in pigs raised in conventional indoor systems, which is similar to the results present in this study. Discrepancies in the frequency of this disease between studies could be explained by the differences in the parasitic control plans adopted in pig farms [38]. However, it is not possible to further speculate on this, considering the lack of information in the treatment plans of the animals prior to arrival at the two slaughterhouses included in this study.

Regarding the conditions affecting the carcasses, skin wounds were the most frequently observed in 167 of 182 examined batches, with a mean within-batch prevalence equal to 6.03 (Table 3). It is generally accepted that the detectability of skin lesions increases after the scalding and dehairing of the carcasses [39] although, in this study, the prevalence of the variable ‘skin wounds’ evaluated postmortem was slightly lower than the prevalence of the variable ‘skin lesions’ evaluated antemortem (9.07%). However, it must be noted that the assessment of the skin antemortem was not only focused on the presence of wounds, but also included other issues such as scars or inflammations.

Dermatitis was also encountered in this study, although the reported prevalence of this condition (2.70%) was much lower compared to that of Maisano et al. [24] (28.03%). The assessment at the slaughterhouse of dermatitis is considered particularly useful with regard to sarcoptic mange [40]. Davies et al. [41] described it as a seasonal effect on the prevalence of popular dermatitis in pigs at the slaughterhouse, with lesions being more prevalent in the autumn–winter period rather than the spring–summer period. Considering the time of the present study, performed during the summer, the lower prevalence of dermatitis could be justified.

Interestingly, erysipelas was not found in this study during antemortem or postmortem inspections, in agreement with the findings of Ceccarelli et al. [12]. On the other hand, Ghidini et al. [10] and Guardone et al. [11] reported erysipelas as the first cause of whole-carcass condemnation in slaughtered pigs, although, in the former study, erysipelas was diagnosed in 0.3% of the total carcasses, while, in the latter study, this percentage was even lower (0.01%). Generally, erysipelas is described as a frequent cause of total condemnation, as also shown in the other studies [6]; however, it is also known that this disease may be affected by several factors, with the environmental temperature described as a critical element for the development of the disease [42]. In fact, Sánchez et al. reported an increased risk of erysipelas in autumn. [6]. Considering that this study was conducted during the summer period, this could justify the non-detection of such a condition, although other factors involved in the development of erysipelas cannot be excluded.

Other conditions, such as cryptorchidism, anemia, muscular alterations, and boar taint were found in none or very few cases; therefore, they were not discussed in this section.

### 4.3. Relationship between Ante- and Postmortem Lesions

In the currently available literature, there are a number of studies reporting data collected at pig slaughterhouses. Most of them described postmortem findings for monitoring purposes [11,12], for comparing different breeding types [9,43], or for meat inspection systems [1,10]. Therefore, little is known about the relationships between ante- and postmortem lesions in pigs, with reference to the capacity of antemortem inspections to predict postmortem outcomes in the same animals. In this study, the total count of postmortem variables increased significantly with the number of observed antemortem lesions, with ‘dirt >30%’ (*p* = 0.0003) and ‘skin lesions’ (*p* = 0.0072) being the most representative variables. Some postmortem variables were positively or negatively related to specific antemortem conditions (Table 4).

The antemortem variable ‘dirt > 30%’ was statistically associated with the postmortem variables ‘respiratory lesions’ (OR= 1.07, 95% CI: 1.02–1.11), ‘skin wounds’ (OR: 1.05; 95% CI: 1.01–1.28), ‘dermatitis’ (OR: 1.11; 95% CI: 1.03–1.20), and ‘kidney lesions’ (OR: 1.18; 95% CI: 1.06–1.32). In this line, it is important to note that these conditions may share on-farm risk factors [13,22]. In particular, the type of floor, the presence of a straw bedding, and a liquid-based feeding system have been identified as risk factors linked to pig dirtiness [22]. These factors may together exacerbate environmental conditions, thereby increasing ammonia level and relative humidity, which are well-known risk factors of respiratory lesions [44]. Moreover, manure on the body can be a possible source of infectious agents, thus increasing the risk of dermatitis [22]. In a study conducted in sow farms, ineffective cleaning of the pens increased the risk of dermatitis from ectoparasites, thereby identifying pen cleanliness as a factor triggering ectoparasites infections [45].

Pen density (number of pigs/pen) and a poorly designed environment can also be included as possible factors involved in the development of the above-described conditions, with particular reference to skin wounds [26]. In fact, these factors can generate a suboptimal social environment, causing excessive fighting and, consequently, the occurrence of skin damage [26]. In this study, information in terms of management and environmental conditions of the farms involved was not collected, except for the variable ‘standardized herd size’; therefore, these aspects cannot be further speculated. Moreover, skin wounds and the dirtiness of the pigs can be affected by the handling before transport, the transport itself, and the lairage; therefore, these conditions need to be analyzed with the due care. Kidney lesions in pigs at the slaughterhouse are linked to several pathogens, such as *Leptospira* spp., porcine coronavirus type 2 (PCV2), and porcine parvovirus (PPV) [46], with *Leptospira* spp. being the most prevalent agent of nephritis in pigs [47]. Several risk factors have been identified for leptospirosis in swine farms [48]. Among these, husbandry practices may play an important role [48]. As aforementioned, husbandry and management data were not in our possession at the time of the study, making it difficult to propose any definitive hypothesis. Relying on the results of this study, it can be suggested that the dirtiness of the pigs may be used as an indicator of suboptimal husbandry practice and, therefore, could trigger the attention of the OV performing PMI. The conditions ‘skin wounds’ and ‘dermatitis’ detected during the postmortem inspection were also related to the variable ‘skin lesions’ recorded during the antemortem inspection. In particular, the overall rates of ‘skin wounds’ and ‘dermatitis’ were similar to the overall results of ‘skin lesions’. These data are in contrast with other findings present in other studies describing that skin lesions are generally more evident after scalding and dehairing of the carcasses; therefore, postmortem inspection has an advantage over antemortem inspection in this matter [25,39]. Moreover, it should be indicated that the evaluation of conditions affecting the skin may be complicated by the presence of manure on the body [24], which was a frequently observed condition in our study. The condition ‘skin lesions’ was also correlated to the postmortem variable ‘milk spot liver’ (OR = 1.24; 95% CI: 0.91–0.97). As previously mentioned, the conditions related to the presence of lesions on the skin, such as wounds or baits, are strictly linked to husbandry and management practices, which are also factors influencing the prevalence and distribution of the parasites responsible for the milk spot livers [49]. The variable ‘standardized herd size’ was instead negatively related with the variable ‘milk spot liver’ (OR = 0.94; 95% CI: 0.91–0.97), suggesting that this condition might be more likely to occur in small farms rather than in large ones. On the contrary, according to Sanchez-Vazquez et al. [49], the frequency of milk spot disease in British slaughterhouses was higher in pigs coming from large (more than 5000 finishers) rather than small (less than 1500 finishers) or medium (between 1500 and 5000 finishers) farms. This discrepancy between results can be justified by the larger number of pigs analyzed by Sanchez-Vazquez et al. [49] compared to ours or by real differences between farms in terms of husbandry practices or management. For instance, smaller farms may provide outdoor access and straw bedding to the pigs, two elements that can predispose to infection with *Ascaris suum* [9].

On the other hand, ‘standardized herd size’ was positively related to the variable arthritis/bursitis (OR = 1.015; 95% CI: 1.004–1.026), which means that pigs coming from larger farms tend to present more arthritis and/or bursitis at the postmortem inspection. Temple et al. [22] described that moderate and severe bursitis tends to occur in pigs raised in conventional farms rather than outdoor farms. At the time of the study, a major part of the farms involved in the data collection were conventional farms in which the floor is usually fully slatted, or in which there is a solid floored lying area combined with a slatted area without bedding. Therefore, this finding can be justified by the type of floor rather than a real effect due to the herd size. Interestingly, a tendency was shown by this study concerning the postmortem variable ‘arthritis/bursitis’ and the antemortem variable ‘lameness’ (*p* = 0.10). In fact, lame animals housed on concrete floors may have a higher risk of bursitis considering the constant pressure on the limbs provided by the body weight on the concrete [22].

A correlation of the antemortem variables ‘lameness’ and ‘ear lesions’ with the postmortem variable ‘abscesses’ was also detected in this study. Abscesses are a common cause of whole-carcass condemnation in pig slaughterhouses [50]. This correlation could be explained by the fact that lame pigs may show an uncontrolled lying-down behavior, a decreased frequency of standing, and an increased frequency of lying postures compared to their pen mates [51]. Therefore, lame pigs can develop lesions due to frequent contact with the floor, which can further evolve in abscesses or be a route for the spread of infections. Moreover, according to Martínez et al. [46], abscesses are frequently found in pigs with growth retardation, and lameness has been shown to be a possible factor of retarded growth in pigs, considering that activity, social interaction, and feeding and drinking behavior are reduced in lame animals [52]. To the best of our knowledge, a direct relationship between ear lesions and abscesses in pig carcasses has not been previously described. The correlation between these variables found in this study could be explained by the fact that lame pigs may be more susceptible to biting behavior on the ears, considering their reduced activity compared to non-lame pigs [53].

## 5. Conclusions

In this study, some conditions evaluated antemortem were predictive of postmortem lesions. In particular, the presence of dirtiness and skin lesions antemortem was shown to be predictive of respiratory lesions, such as pneumonia and pleurisy, skin wounds, dermatitis, and kidney lesions, while lameness was correlated to the presence of abscesses in the carcass and not significantly to the presence of arthritis/bursitis. This study supports, on one hand, the implementation of PMI procedures in pigs on the basis of evidence gained from AMI, while, on the other hand, it sustains the crucial importance of feedback from data collected at the abattoir provided to pig farmers. In fact, these data can be used to improve the management and husbandry practices at a farm level. The integration of further farming parameters, such as management procedures, into the scheme would make it more reliable and advantageous toward a risk-based approach to meat inspection.

## Figures and Tables

**Table 1:** List of the conditions and the guidelines adopted in this study (partially adapted from [10]). AM: antemortem; PM: postmortem.

Condition/Lesions	Guideline	AM/PM
Dirt >30%	Detection of the presence of manure on the body covering 30% or more of the surface of a side.	AM
Skin lesions	Detection of at least one deep lesion or multiple bruises or injuries due to mismanagement during loading/unloading of a side of the body.	AM
Ear lesions	Detection of the presence of bruises and injuries or outcomes of wounds such missing ear parts or ear necrosis.	AM
Lameness	Detection of the pig being severely lame with minimum weight-bearing on the affected limb.	AM
Umbilical/inguinal hernia	Detection of the presence of umbilical or inguinal hernia.	AM
Suppressed	Detection of the pig requiring suppression in the truck or in the pen due to severe lesions or injuries.	AM
Dead on arrival	Detection of dead pigs during transport.	AM
Non-walking	Detection of the pigs being unable to walk with no weightbearing on the affected limb.	AM
Tail lesion	Detection of a presence of fresh blood, inflammation, infection, or missing part of tail tissue.	AM
Dyspnea	Detection of pigs with difficulty breathing.	AM
Anemia	Detection of carcasses with pale skin and mucosae.	AM
Cachexia	Detection of pigs with poor body condition.	AM
Erysipelas	Detection of typical skin lesions.	AM
Pleurisy	Detection of adhesions present on the carcass and/or fibrin present on the visceral layer of the pleura.	PM
Pneumonia	Detection of pneumonia and the outcomes of pneumonia. Detection of pneumonia when an entire lobe is involved or when the lesion involves two contralateral lobes. Lung abscesses (even one) are considered as pneumonia.	PM
Pericarditis	Detection of fibrin on the heart surface.	PM
Myocarditis	Detection of acute/chronic myocarditis.	PM
Milk spot liver	Detection of typical milk spot lesions.	PM
Liver alterations	Detection of hepatitis and outcomes of hepatitis. The presence of fibrin on the capsule should be classified as peritonitis. Detection of steatosis and liver necrosis.	PM
Liver cirrhosis	Record of liver affected by extended scar tissue and cirrhosis.	PM
Dermatitis	Detection of a thickening of the skin. Detection of lesions exceeding 50% of the body surface or less when confined to the abdominal region and chest. Detection of carcasses massively affected by bites of ectoparasites and dermatitis.	PM
Cryptorchidism	Detection of testicles in abdomen.	PM
Skin wounds	Wounds from intraspecific fights and numerous injuries that deepen into the derma, with possible infection.	PM
Arthritis/bursitis	Record of at least one bursa and/or inflamed joint.	PM
Stomach repletion	Record of not-emptied stomach.	PM
Nephrosis/hydronephrosis	Detection of degenerative process of the kidneys.	PM
Interstitial nephritis	Record of interstitial nephritis.	PM
Abscesses	Detection of abscesses that are not located in the lung or in the liver. Also Detection of phlegmon as abscesses.	PM
Enteritis/colitis	Record of thickening of the small intestine, with or without hemorrhages or necrosis.	PM
Splenomegaly	Detection of more than 50% of the organ affected.	PM
Peritonitis	Report of inflammation and/or infection of the peritoneum.	PM
Generalized lymphadenitis	Detection of an increased volume of lymph nodes in the carcass.	PM
Umbilical/inguinal hernia	Detection of umbilical or inguinal hernia.	PM
Intestinal ascariasis	Record of the presence of ascariasis at intestinal level.	PM
Jaundice	Record of the presence of generalized jaundice.	PM
Muscular lesion/color alteration	Detection of any change in the color or degenerative process on the carcasses.	PM
Tail lesion	Detection of inflammation, infection, or missing part of tail tissue.	PM
Gastroesophageal ulcer	Detection of hemorrhages or ulcer at gastric level.	PM
Boar taint	Report of carcasses with pungent and abnormal odor.	PM
Purulent spondylitis	Report of abscesses in the vertebral bodies.	PM
Neoplastic process	Record of any tumor present in the carcass regardless of size and distribution.	PM
Edema (pancreatic)	Detection of edema at pancreatic level.	PM
Erysipelas	Detection of typical skin lesions.	PM
Anemia	Detection of carcasses with pale skin and mucosae.	PM
Cachexia	Detection of carcasses with poor body condition.	PM
Insufficient bleeding	Record of intense congestion of the head and neck area.	PM
Goiter	Report of any swelling in the neck due to an enlarged thyroid gland.	PM

**Table 2:** Number of positive batches and within-batch prevalence of recorded antemortem lesions in slaughtered pigs (*N* = 182 batches).

Variable	No. of Positive Batches	Within-Batch Prevalence (%)		
		Mean	95% CI	Range (Min–Max)
Dirt >30%	177	37.13	33.85–40.41	0–92.31
Skin lesions	167	9.07	7.83–10.32	0–57.78
Ear lesions	106	3.30	2.47–4.14	0–55.38
Lameness	52	0.30	0.22–0.39	0–4.23
Umbilical hernia	37	0.25	0.16–0.34	0–3.20
Suppressed	23	0.11	0.05–0.17	0–3.01
Tail-biting	19	0.11	0.07–0.16	0–1.54
Non-walking	19	0.09	0.05–0.14	0–1.54
Dead on arrival	7	0.04	0.01–0.07	0–2.22
Dyspnea	3	0.01	0–0.03	0–0.77
Anemia	0	0	0	0
Cachexia	0	0	0	0
Erysipelas	0	0	0	0

**Table 3:** Number of positive batches and within-batch prevalence of recorded postmortem lesions in slaughtered pigs (*N* = 182 batches).

Variable	No. of Positive Batches	Within-Batch Prevalence (%)		
		Mean	95% CI	Range (Min–Max)
Pleurisy	181	17.21	16.00–18.43	0–49.29
Pericarditis	177	7.82	7.03–9.30	0–35.23
Pneumonia	171	8.16	6.40–7.15	0–57.78
Skin wounds	167	6.03	5.34–6.71	0–21.60
Milk spot liver	151	7.60	5.97–9.23	0–100
Liver alterations	153	4.89	4.15–5.62	0–29.23
Dermatitis	133	2.70	2.18–3.21	0–28.52
Arthritis/bursitis	125	1.59	1.33–1.85	0–11.11
Stomach repletion	112	5.35	4.05–6.64	0–76.92
Nephrosis/Hydronephrosis	66	1.33	0.98–1.68	0–11.11
Abscesses	60	0.91	0.76–1.07	0–6.00
Enteritis/colitis	51	0.52	0.32–0.71	0–13.64
Interstitial nephritis	50	1.26	0.87–1.66	0–12.77
Splenomegaly	40	0.29	0.18–0.40	0–5.26
Peritonitis	16	0.11	0.03–0.18	0–4.44
Cryptorchidism	12	0.07	0.01–0.13	0–4.41
Umbilical/inguinal hernia	10	0.04	0.01–0.06	0–1.41
Intestinal ascariasis	9	0.28	0.04–0.52	0–12.41
Jaundice	5	0.02	0–0.03	0–0.78
Muscular lesions/color alterations	2	0.01	0–0.02	0–0.77
Tail-biting	2	0.01	0–0.01	0–0.70
Gastroesophageal ulcer	1	<0.01	0–0.01	0–0.82
Boar taint	1	<0.01	0–0.01	0–0.77
Liver cirrhosis	1	<0.01	0–0.01	0–0.71
Purulent spondylitis	1	<0.01	0–0.01	0–0.70
Neoplastic processes	1	<0.01	0–0.01	0–0.27
Edema (pancreatic)	0	0	0	0
Erysipelas	0	0	0	0
Death before stunning	0	0	0	0
Anemia	0	0	0	0
Cachexia	0	0	0	0
Insufficient bleeding	0	0	0	0
Goiter	0	0	0	0
Generalized lymphadenitis	0	0	0	0
Myocarditis	0	0	0	0

**Table 4:** Minimal selected models exploring the relationship between the proportion of postmortem and antemortem lesions observed in slaughtered pig batches (*N* = 182). In all models, farm was included as a random factor.

Response Variable	Explanatory Variables	Parameter Estimate (± SE)	χ^2^_1_	*p*-Value
Respiratory diseases	Dirt >30%	0.65 ± 0.21	9.58	0.0020
	Ear lesions	1.37 ± 0.78	3.04	0.081
	Lameness	12.5 ± 8.15	2.34	0.13
Pericarditis	Dirt >30%	0.36 ± 0.21	2.87	0.090
Skin wounds	Skin lesions	1.31 ± 0.60	4.67	0.0308
	Dirt >30%	0.51 ± 0.25	4.01	0.0453
	Suppressed	−30.8 ± 20.0	2.38	0.12
Milk spot liver	Standardized herd size	−0.54 ± 0.15	13.54	0.0002
	Skin lesions	2.16 ± 0.93	5.41	0.020
	Dirt >30%	0.65 ± 0.44	2.16	0.15
Liver alterations	Skin lesions	1.40 ± 0.78	3.21	0.073
	Ear lesions	2.04 ± 1.16	3.12	0.077
Dermatitis	Dirt >30%	1.15 ± 0.39	8.73	0.0031
	Skin lesions	2.96 ± 0.69	18.52	0.0002
	Standardized herd size	0.008 ± 0.11	0.01	0.94
Arthritis/bursitis	Standardized herd size	0.13 ± 0.05	7.42	0.0064
	Lameness	17.91 ± 10.98	2.66	0.10
	Dirt >30%	0.53 ± 0.35	2.27	0.13
Kidney lesions	Dirt >30%	1.69 ± 0.54	9.76	0.0018
	Umbilical hernia	26.6 ± 16.7	2.55	0.11
Abscesses	Ear lesions	2.81 ± 0.98	8.25	0.0041
	Lameness	35.18 ± 10.32	11.60	0.0007

## Data Availability

Data are available on request due to privacy reasons.
